# An Intersectional Exploration of Sexual Lifestyles and Health Across the Life Course in France

**DOI:** 10.1111/psrh.70075

**Published:** 2026-08-21

**Authors:** Caroline Moreau, Aline Bohet, Laurent Rigal, Claire Sauvage, Florence Lot

**Affiliations:** ^1^ INSERM, Soins Primaires et Prévention, CESP Centre for Research in Epidemiology and Population Health Villejuif France; ^2^ Department of Population, Family and Reproductive Health Johns Hopkins University Bloomberg School of Public Health Baltimore Maryland USA; ^3^ Direction Des Maladies Infectieuses, Santé Publique Saint‐Maurice France

## Abstract

**Introduction:**

The relationship between sexuality and health is complex, with both beneficial and detrimental aspects. We examined how these associations varied by age, gender, social condition, and disability among adults in France.

**Methods:**

This study draws on the CSF‐2023 national survey on sexuality in France. Our analytic sample comprised 19,192 sexually active respondents aged 18–89 years. We used multivariable logistic regressions to examine associations between dimensions of sexuality (sexual activity in the past year, frequency of sex, penetrative sex, masturbation, lifetime sexual violence, sexual dysfunction, and sexual satisfaction) and three health outcomes (self‐perceived health, depression, and anxiety). We tested for interactions by gender, sexual identity, partnership status, economic hardship, and disability/chronic illness.

**Results:**

Over a quarter of respondents reported poor/fair health and 18% experienced symptoms of depression or anxiety. Sexual dissatisfaction and past sexual violence increased the likelihood of poor/fair health, depression, and anxiety for both genders. Other associations varied by sexuality indicator, health condition, and age. Younger respondents experienced more negative health impacts from sexual violence, while sexual inactivity was more strongly linked to depression among men over 60 years and anxiety among women over 60 years. Additionally, masturbation was only associated with decreased odds of anxiety among women over 60 years.

**Conclusions:**

This study underscores the intricate relationship between sexuality and health, uncovering varying positive correlations across the life course. Considering the strong connections between health and sexual satisfaction, quality of sexual life should be part of clinical assessments as an integral component of health and well‐being.

## Abstract (fr)

**Introduction:**

La relation entre la sexualité et la santé est complexe, comportant à la fois des aspects bénéfiques et néfastes. Nous avons examiné comment ces associations variaient selon l'âge, le genre, la condition sociale et le handicap.

**Méthodes:**

Cette étude s'appuie sur l'enquête nationale CSF‐2023 sur la sexualité en France. Notre échantillon comprenait 19,192 répondants sexuellement actifs âgés de 18 à 89 ans. Nous avons utilisé des régressions logistiques multivariées pour examiner les associations entre les dimensions de la sexualité (activité sexuelle dans les 12 mois, fréquence des rapports sexuels, rapports pénétratifs, masturbation, antécédents de violences sexuelles, dysfonctionnements sexuels et satisfaction sexuelle) et trois indicateurs de santé (santé perçue, dépression et anxiété). Nous avons testé les interactions selon le genre, l'identité sexuelle, le statut partenarial, la précarité économique et le handicap/maladie chronique.

**Résultats:**

Plus d'un quart des répondants ont déclaré avoir une santé médiocre ou mauvaise et 18% ont éprouvé des symptômes de dépression ou d'anxiété. L'insatisfaction sexuelle et les violences sexuelles étaient associées à une probabilité plus élevée de mauvaise santé perçue, de dépression et d'anxiété pour les deux sexes. D'autres associations variaient selon l'indicateur de sexualité, la condition de santé et l'âge. Les violences sexuelles avaient un impact plus négatif sur la santé des jeunes, tandis que l'inactivité sexuelle était plus fortement liée à la dépression chez les hommes de plus de 60 ans et à l'anxiété chez les femmes de plus de 60 ans. De plus, la masturbation était associée à une diminution des risques d'anxiété chez les femmes de plus de 60 ans.

**Conclusions:**

Cette étude souligne la relation complexe entre la sexualité et la santé, révélant diverses corrélations positives tout au long de la vie. Compte tenu des liens étroits entre la santé et la satisfaction sexuelle, la qualité de la vie sexuelle devrait faire partie des évaluations cliniques en tant qu'élément intégral de la santé et du bien‐être.

## 1 Introduction

The World Health Organization (WHO) acknowledges the critical role of sexuality in human well‐being [1]. More broadly, existing research suggests that the relationship between sexuality and health is complex and bidirectional: sexuality can affect physical and mental health, while health status can also shape sexual experiences and functioning [2]. On the one hand, extensive research highlights the health risks associated with unprotected or non‐consensual sexual activity. These include sexual and reproductive health (SRH) consequences, such as sexually transmitted infections (STIs) [3, 4] and maternal deaths from unintended pregnancies [5], as well as broader physical [6] and mental health consequences [4, 7, 8, 9] that may persist across the life course. Conversely, medical conditions can negatively affect sexuality, with studies indicating reduced sexual activity and functioning among individuals with chronic illnesses, including cancer [10], cardiovascular disease [11], or mental health issues [12]. Cross‐sectional surveys conducted among midlife and aging populations indicate that individuals in poor health are less likely to engage in sexual activity, experience less frequent sexual encounters, and are less satisfied with their sexual lives [13, 14], with some variations by gender [15]. These observations extend to younger populations [16, 17, 18], with varying effects by type of sexual practice [19]. These findings underscore that sexuality and health are mutually linked, rather than operating as separate domains. They also point to the relevance of considering gender in understanding these associations.

At the same time, sexuality is not only a source of disease burden [20]; a satisfying sexual life may also confer health benefits [2, 21]. In a 2013 review, Anderson highlights the positive correlations between sexual satisfaction and both physical and mental health [21]. One proposed pathway is through improved self‐esteem, a key contributor to overall well‐being. Other suggested biomedical mechanisms include enhanced immune function, aiding in the prevention of infection [22, 23]; improved cardiovascular health, as demonstrated by lowered heart rate and blood pressure [24]; and stress relief through increased oxytocin release [25]. Longitudinal studies provide the strongest evidence for a positive influence of sexuality on health. For example, the English Longitudinal Study of Aging found that declines in sexual desires or sexual activity among older populations predict worsening self‐rated health and increasing incidence of chronic disease, including cancer and cardiovascular disease, over time [26]. Likewise, a longitudinal study in Germany reported that sexual satisfaction in middle‐aged adults was associated with better subsequent self‐reported health and lower physical illness [27]. Another study in the United States (US) found that a higher frequency of sexual activity lowered the risk of all‐cause, cardiovascular, and cancer mortality [28].

Taken together, these findings suggest that the relationship between sexuality and health is bidirectional: health shapes sexual life, but sexual life may also shape subsequent health and well‐being. Yet this interrelationship is unlikely to take the same form at all ages because sexuality is experienced within changing bodies, relations, and normative contexts across the life course. Much of the existing literature, however, lacks a life course perspective and therefore overlooks these shifting dynamics [29]. A better understanding of how the links between sexuality and health evolve across the life course is critical for tailoring sexual health promotion efforts to the changing needs of individuals.

Recognizing this life course dimension also helps to clarify an important limitation in the literature. Public health research has often emphasized the negative outcomes of sexuality in younger populations while highlighting its positive aspects in aging populations. This imbalance reflects not only age‐related changes in sexual activity and health, but also the medicalization of sexuality [30], which tends to focus on pregnancy and STIs in younger individuals and on sexual functioning or enhancement in older adults. Such a framing limits our understanding of the positive effects of sexuality on the health of younger individuals while also failing to address sexual risks faced by older populations.

Beyond the life course perspective, research on the association between sexuality and health should also situate this relationship within the social contexts that shape people's lives [31]. Gender is especially important because it influences both sexuality [31] and health [32] and may therefore shape the relationship between them in distinct ways for women and men. For example, lower sexual frequency has been linked to higher subsequent mortality among women, but not consistently among men [33]. More broadly, a substantial body of research has shown that sexuality and sexual health are shaped by intersecting social hierarchies, including gender, sexual orientation, social class, race/ethnicity, or migration status [34]. However, intersectional approaches have mostly been used to investigate sexual risks, with less attention to a broader spectrum of correlations, including associations between sexuality and general health across the life course.

A long tradition of population‐based research on sexuality in France sheds light on gender and social disparities in various aspects of sexuality (norms, relationships, and behaviors) and sexual health outcomes, including STIs, unintended pregnancies, and sexual violence [31, 35]. However, the role of these intersecting factors in the correlations between sexuality and broader aspects of health has not been explored. Figure 1 provides a simplified conceptual summary of the relationships outlined above and situates the proposed study. It illustrates the bidirectional links between multiple dimensions of sexuality and physical and mental health, while highlighting that these associations change across the life course and are embedded in broader social contexts. It also emphasizes the central role of gender, in intersection with other social hierarchies, in shaping sexuality, health, and the links between them.

**FIGURE 1:**
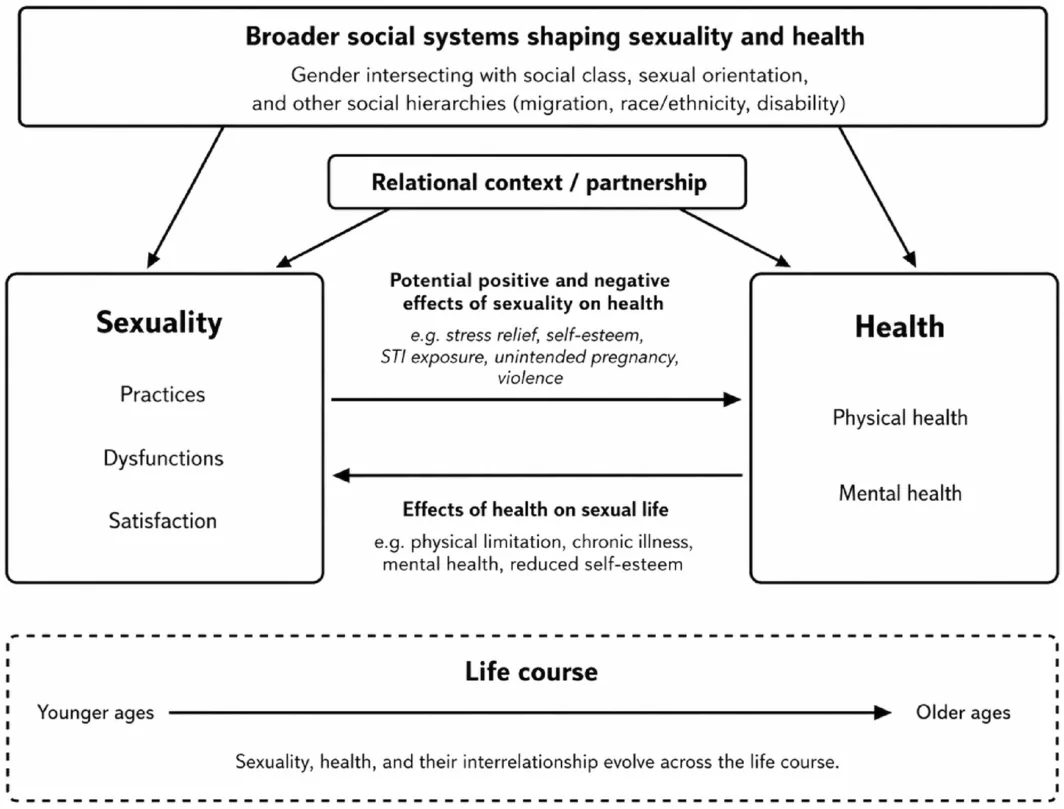
A conceptual understanding of the bidirectional association between sexuality and health.

Building on this framework, we sought to investigate the interplay between sexuality and self‐reported general health and mental health across the life course. Specifically, our objectives were to examine the associations between different dimensions of sexuality (practices and subjective experiences) and self‐reported general and mental health; to assess whether these associations differed by gender and across life stages; and to evaluate whether they varied according to sexual identity, partnership status, and economic hardship.

## 2 Methods

### 2.1 Study Design and Procedures

CSF‐2023 employed a national probability sample comprising 21,259 respondents aged 15 to 89 years in continental France, selected through random digit dialing, with only half of the eligible adults aged 65–89 included due to cost constraints. The response rate was 34%. The initial sample underrepresented adolescents aged 15–17, partly because of challenges in reaching parents to obtain consent by phone, and individuals with less than a high school diploma, who are generally less likely to participate in surveys. However, the sample closely matched census benchmarks for regional distribution and household composition [36]. Among the 21,066 adult respondents (18–89 years) who were eligible for Part 2 of the survey, the distribution of the 12,966 individuals who completed Part 2 was within a 2‐percentage point margin of the eligible sample for all indicators except education and employment status. We therefore applied post‐stratification weights to the full sample (*N* = 21,259) and inverse probability weights to the Part 2 subsample (*n* = 12,966) to account for these distortions. A more detailed examination of non‐response bias and weighting procedures is published in Moreau et al. 2026 in this special issue [36].

Part 1 of the data collection, conducted by trained interviewers via phone between November 2022 and November 2023, lasted 33 min, while Part 2 took 30 min to self‐complete online. Part 1 covered sociodemographic information, sexual attitudes, sexual relationships, and sexual practices, as well as SRH topics, self‐rated health, disability, and chronic illness. Part 2 gathered information on mental health, along with other topics such as first and last sexual encounters, digital sexual practices, and utilization of SRH services.

Participants provided oral consent to participate in each part of the study after being presented with the goals, procedures, and potential risks associated with study participation. Minors aged 15–17 who only participated in Part 1 obtained parental consent and provided their assent to participate. The study received ethical approval from the Committee of Protection of People South East 1 (CPP Sud Est 1), the Inserm Ethics Evaluation Committee (CEEI‐IRB), and the Commission Nationale de l'Informatique et des Libertés (CNIL).

For this analysis, we selected 20,304 respondents aged 18–89 years who had ever reported any sexual practice (penetrative or non‐penetrative), of whom 19,192 had complete data on perceived health. The sample closely mirrored the original distribution, except for gender and residence, with a greater representation of women and city dwellers. Subsamples of 11,904 and 11,947 who responded to Part 2 had complete data on depression and anxiety, respectively.

### 2.2 Measures

We defined three sets of indicators, treating domains of sexuality as key independent variables, health as primary outcome variables, and gender, partnership status, sexual identity, disability/chronic illness status, and economic hardship as confounders or moderators of the relationships between sexuality and health outcomes. Specifically, we identified seven indicators of sexuality, including any sexual intercourse in the last 12 months (yes/no), penetrative sexual practices in the past 12 months (yes/no), masturbation in the past 12 months (yes/no), number of sexual acts in the last 4 weeks, history of sexual violence, sexual dysfunction causing distress, and sexual satisfaction. We defined sexual dysfunction based on a set of questions exploring issues related to libido, pain during intercourse, and lack of pleasure for all respondents, as well as vaginal dryness for women and erectile dysfunction for men [37]. We considered respondents who indicated that any of these issues lasted at least 6 months and were a cause of distress for their sexuality to have sexual dysfunction.

Our key health indicators consisted of respondents' self‐perceived health (very good/good/fair/bad), as well as depression and anxiety ascertained by the Patient Health Questionnaire‐9 (PHQ‐9) and Generalized Anxiety Disorder‐7 (GAD‐7) scores [38, 39]. We included self‐rated health because it is a widely used and validated global indicator of health status in population‐based research [40], with strong evidence of predictive validity for morbidity and mortality [41, 42].

Our measures of mental health, PHQ‐9 and GAD‐7 are also widely used in population‐based research, and the scales had good internal reliability in our sample (Cronbach alpha = 0.87 and 0.90, respectively). We summarized the measures into two scores, further dichotomized at a cutoff point of 10 corresponding to moderate and severe forms of depression or anxiety [38, 39]. Perceived health was available for all respondents, while PHQ‐9 and GAD‐7 scores were only available for respondents who completed Part 2 of the survey.

We considered a range of sociodemographic characteristics: gender, age, education, economic hardship, type of health insurance plan, migration, and residence and health conditions, including functional disability, chronic illness, partner disability, and uro‐gynecological conditions (prostate‐related problems for men and problems related to the breast, ovaries, and uterus for women). We asked respondents whether they identified as men, women, or another category. Because the wording of the question in French does not clearly distinguish between sex and gender, and five respondents selected another category, we use the term gender throughout, while acknowledging that this measure remains ambiguous. Migration was based on country of birth and nationality at birth, distinguishing respondents born in France from those born in a foreign country. We classified respondents as having experienced economic hardship if they indicated that they had faced a difficult financial situation or couldn't make ends meet. We also asked them if they had been limited in their daily activities for at least 6 months due to a health problem and if they had a chronic illness or health problem that has lasted for at least 6 months to ascertain functional disability and chronic illness. Less than 0.5% of data were missing for sociodemographic, disability, and chronic illness, but 10% were missing for financial difficulty due to a skip pattern error that was corrected during data collection. We therefore imputed missing data on “financial difficulty” using predicted values of a multivariate logistic regression model. We also checked that the analysis among the sample with complete data yielded similar results.

### 2.3 Analysis

Following the presentation of the sample population, we analyzed the distributions of sexuality measures and health indicators based on sociodemographic factors and functional disability/chronic health status. Next, we examined bivariate associations between sexuality measures and each health outcome and conducted multivariate regression models adjusting for all covariates (sociodemographic, functional disability/chronic illness). Finally, we performed stratified analysis by age (18–29, 30–59, 60–89 years), current partnership status, sexual identity, health status (functional disability or chronic illness), and economic instability. We defined age groups to ensure sufficient sample size for each age category and capture key life‐course stages relevant to sexuality and health, including partnership and family formation, mid‐adulthood, when conjugal, parental, and work‐related roles are often most salient, and later‐life characterized by changes in health, retirement, and partnership status. We tested for interactions by sexual identity, partnership status, and economic hardship in multivariate regression models. We weighted all analyses to account for unequal probability of selection and non‐response and conducted all analysis in Stata17.

## 3 Results

### 3.1 Sample Characteristics

We detail the characteristics of the sample in Table 1. The average age of respondents was 50.2 years, and 72% had children. Respondents were evenly distributed across large cities, medium/small cities, and rural areas. The vast majority (93%) were born in France. More than a third of the sample (34%) had some college education, and a significant percentage (40%) faced financial difficulty. More women than men reported a functional disability (31% versus 24%), and an equal share of women and men (16%) had a partner with a functional disability.

**TABLE 1:** Characteristics of the CSF2023 sample population of adults aged 18–89 years living in continental France (*N* = 19,192).

		Men *n* = 9356	Women *N* = 9836
		%	%
Age	18–29	16.2	15.6
	30–39	16.8	16.1
	40–49	16.8	16.3
	50–59	19.0	18.6
	60–69	16.5	16.4
	70–89	14.7	17.1
Current partner		77.1	74.9
Children		67.9	76.5
Education	< High school graduation	50.1	43.0
	High school graduation	18.2	20.2
	Bachelor	20.0	24.4
	Graduate level	11.6	12.4
Facing financial difficulty		37.5	40.9
Health insurance	Social security + private insurance	88.0	90.1
	Social security alone	4.9	3.6
	Low‐income public coverage or no coverage	5.8	5.6
	Other	1.4	0.7
Migration	Foreign born	7.7	7.0
Residence	Large city/suburb	27.2	26.9
	Medium or small size city	36.7	37.8
Functional disability^a^		24.1	30.5
Chronic illness^b^		42.1	48.9
Uro‐gynecological condition^c^		1.8	17.9
Partner disablity^d^		15.3	16.4
Sexual self‐identity	Heterosexual	92.2	91.2
	Non heterosexual	4.5	3.9
	Undefined	3.4	4.9
History of sexual violence	None	87.1	62.7
	Unwanted sexual contact	6.3	14.4
	Sexual assault (attempted or forced sex)	6.7	22.9
Number of sexual partners in the last year	0	21.7	27.9
	1	64.2	64.3
	> 1	14.2	7.8
Penetrative sex last 12 months*		91.7	93.2
Masturbation last 12 months		64.9	44.5
Sexual dysfunction in last 12 months^*e*^		4.8	8.3
Sexual satisfaction	Very satisfied	37.6	44.6
	Somewhat satisfied	40.8	38.1
	Unsatisfied	21.6	17.3

* Among respondents who reported any sexual activity in the last 12 months.

^a^ Functional disability: limitations in daily activities for at least 6 months due to a health problem.

^b^ Chronic illness: chronic illness or health problem lasting at least 6 months.

^c^ Uro‐gynecological condition: prostate‐related problems in men and breast‐, ovary‐, or uterus‐related problems in women.

^d^ Partner disability: partner reported to have limitations in daily activities for at least 6 months due to a health problem.

^e^ Sexual dysfunction (last 12 months): at least one sexual problem lasting 6 months or more and causing distress.

A quarter of respondents (75%) had a current partner, and 92% identified as heterosexual. Thirty‐seven percent of women and 13% of men reported a history of sexual violence. Three‐quarters of respondents (76%) were sexually active in the past year, and 65% of men and 45% of women reported masturbating in the past year. Sexually active respondents in the past year had, on average, 5.3 acts of intercourse in the past four weeks. Men were more dissatisfied with their sexual lives than women (22% versus 17%), but more women reported sexual dysfunction causing distress in the last year (8% versus 5%).

### 3.2 Link Between Sexuality/Sexual Health and Health Outcomes

A little over a quarter of respondents reported poor or fair health, 18% had depressive and anxiety symptoms (Table 2). Women were significantly more likely than men to report fair or poor health (29% vs. 25%) as well as depressive or anxiety symptoms (22% and 24% vs. 14% and 12%, respectively). Perceptions of fair or poor health, depression, and anxiety increased with economic difficulty (perceived financial difficulty, government health plan for low‐income residents), among respondents without partners, and those with a functional disability or a chronic health condition. Conversely, most educated participants were less likely to report poor/fair health, depression, and anxiety (Table 2). Fair or poor health increased with age and among individuals with children, while the reverse was true for depression and anxiety. Finally, migration was unrelated to health conditions, while place of residence was inconsistently related to health conditions.

**TABLE 2:** Percentage of CSF‐2023 participants (*N* = 19,192) perceiving poor/fair health, depression, or anxiety according to sociodemographic and sexuality/sexual health indicators stratified by gender.

		Men			Women		
		Poor/fair health	Depression	Anxiety	Poor/fair health	Depression	Anxiety
*N*		9356	5538	5559	9836	6366	6388
Total		24.5	14.0	12.0	29.3	22.3	23.5
Age	18–29	**12.9**	**29.5**	**16.7**	**16.6**	**37.4**	**33.5**
	30–39	**13.1**	**15.0**	**13.3**	**20.9**	**24.3**	**24.5**
	40–49	**30.6**	**12.4**	**14.6**	**26.1**	**19.6**	**21.3**
	50–59	**29.8**	**12.8**	**11.2**	**31.7**	**18.6**	**22.6**
	60–69	**31.0**	**9.8**	**8.0**	**34.5**	**15.0**	**19.3**
	70–89	**40.8**	**7.0**	**9.3**	**44.5**	**19.7**	**20.0**
Functional disability^a^	Yes	**60.1**	**21.5**	**17.5**	**66.9**	**33.2**	**32.8**
	No	**13.7**	**11.5**	**10.1**	**13.5**	**17.7**	**19.5**
Chronic disease^b^	Yes	**45.6**	**17.2**	**14.1**	**49.7**	**27.8**	**27.4**
	No	**9.4**	**11.4**	**10.3**	**10.2**	**16.9**	**19.7**
Uro‐gynecological condition^c^	Yes	**38.0**	10.0	13.0	**37.9**	24.7	25.9
	No	**24.2**	14.1	11.9	**27.4**	21.4	22.6
Current partner	Yes	**21.9**	**10.4**	**10.7**	**25.8**	**20.3**	22.3
	No	**30.3**	**26.3**	**15.3**	**37.4**	**27.3**	24.9
Partner Functional disability^d^	Yes	**35.2**	13.8	**15.9**	**39.3**	23.8	**27.4**
	No	**22.5**	14.0	**11.2**	**27.4**	22.0	**22.8**
Children	Yes	**25.9**	**10.6**	**11.0**	**31.2**	**18.9**	**21.6**
	No	**21.5**	**21.4**	**14.2**	**23.2**	**32.8**	**29.2**
Education	< High school	**32.2**	**14.7**	**13.2**	**40.7**	**23.0**	**25.2**
	High school	**18.3**	**16.1**	**11.3**	**25.2**	**28.1**	**27.6**
	Bachelor	**18.0**	**12.7**	**11.8**	**21.1**	**19.4**	**19.9**
	Graduate	**12.4**	**10.0**	**8.1**	**13.3**	**16.6**	**18.6**
Financial difficulty	Yes	**34.6**	**20.7**	**16.2**	**39.6**	**30.2**	**31.1**
	No	**18.4**	**10.0**	**8.8**	**22.4**	**16.7**	**16.4**
Health insurance	National + private	**23.1**	**12.8**	**10.9**	**28.4**	**21.5**	**22.8**
	National only	**26.4**	**21.1**	**26.7**	**31.0**	**26.1**	**21.5**
	Low‐income public coverage or no coverage	**44.0**	**28.8**	**19.0**	**44.4**	**34.6**	**37.2**
	Other	**24.4**	**16.8**	**13.2**	**29.9**	**25.2**	**37.2**
Migration	Foreign born	27.7	14.0	7.6	30.0	23.0	21.8
	Born in France	24.2	13.5	12.3	29.3	22.3	23.6
Residence	Large city/suburb	**21.6**	15.4	12.3	27.7	**23.7**	25.2
	Medium or small size city	**27.5**	14.5	10.2	28.8	**23.8**	23.6
	Rural	**24.5**	12.4	13.5	29.3	**19.7**	22.0
Sexual identity	Heterosexual	**24.2**	**13.2**	11.6	**29.0**	**20.8**	**22.4**
	Non‐heterosexual	**26.0**	**27.3**	13.9	**30.6**	**43.3**	**37.7**
	Undetermined	**46.9**	**18.3**	19.3	**47.3**	**31.5**	**34.3**
Sex last 12 months	Yes	**21.2**	**12.8**	11.5	**24.5**	**20.9**	**22.3**
	No	**40.8**	**19.4**	14.0	**44.0**	**26.8**	**27.2**
Masturbation last 12 months	Yes	**20.1**	**16.4**	**13.7**	**22.9**	23.3	**21.7**
	No	**32.6**	**8.8**	**8.4**	**34.4**	21.4	**25.2**
Penetrative sex last 12 months*	Yes	**20.0**	12.6	11.7	23.7	21.1	22.3
	No	**31.4**	16.3	9.0	28.2	14.6	18.9
Number of sexual acts last 4 weeks*	0–4	**25.1**	**15.0**	**13.0**	**27.8**	21.8	**24.1**
	5–8	**15.8**	**8.2**	**7.7**	**19.2**	18.2	**18.3**
	> 8	**15.1**	**10.8**	**11.4**	**19.8**	20.8	**20.9**
Sexual dysfunction in last 12 months^e^	Yes	**37.1**	**20.9**	**22.8**	31.4	**29.9**	**29.9**
	No	**23.8**	**13.6**	**11.4**	29.1	**21.6**	**22.9**
History of sexual violence	No	24.0	**12.3**	**10.7**	**27.5**	**17.8**	**20.2**
	Unwanted sexual contact	27.3	**19.9**	**18.0**	**33.9**	**25.8**	**28.7**
	Sexual assault	27.8	**29.0**	**21.1**	**31.5**	**31.6**	**28.6**
Sexual satisfaction	Very satisfied	**17.6**	**9.8**	**10.0**	**24.2**	**18.4**	**19.9**
	Somewhat satisfied	**22.8**	**10.8**	**9.3**	**30.1**	**21.4**	**23.1**
	Unsatisfied	**39.8**	**25.6**	**19.6**	**40.8**	**34.3**	**33.2**

*Note:* In bold: significant differences at *p* < 0.05.

* Number of sexual acts in the last 4 weeks and type of sexual acts are compiled among respondents who indicated being sexually active in the last 12 months.

^a^ Functional disability: limitations in daily activities for at least 6 months due to a health problem.

^b^ Chronic illness: chronic illness or health problem lasting at least 6 months.

^c^ Uro‐gynecological condition: prostate‐related problems in men and breast‐, ovary‐, or uterus‐related problems in women.

^d^ Partner disability: partner reported to have limitations in daily activities for at least 6 months due to a health problem.

^e^ Sexual dysfunction (last 12 months): at least one sexual problem lasting 6 months or more and causing distress.

Results indicate notable variation in the three health indicators of interest according to sexuality and sexual health. Sexual dissatisfaction was consistently associated with rising reports of poor/fair health, as well as depressive and anxiety symptoms. Sexual dysfunction, sexual inactivity in the last 12 months, and fewer acts of sexual intercourse (less than 5) in the past 4 weeks also mostly correlated with worse health conditions for both genders with a few exceptions: sexual dysfunction was unrelated to poor/fair for women, sexual inactivity was unrelated to anxiety for men, and frequency of sex was unrelated to depression for women. Likewise, respondents who self‐identified as non‐heterosexual (homosexual, bisexual, or other) reported worse health conditions except for anxiety in men. Other associations were gender or condition‐specific, such as masturbation associated with more mental health symptoms but better general health among men, with the latter association also observed among women.

In multivariate analysis adjusted for sociodemographic characteristics, disability, and chronic conditions (Table 3), sexual dissatisfaction remained strongly associated with increased odds of reporting poor/fair health, depressive symptoms, and anxiety symptoms among both genders. Similarly, respondents with a history of sexual violence (unwanted contact or assault) exhibited higher odds of poor/fair general and mental health conditions, except for men's general health. Other associations varied by condition, gender, or both. For example, sexual activity reduced the odds of all health conditions among women, but not among men. Masturbation in the past year was linked to decreased odds of poor/fair health for both genders and was associated with lower odds of anxiety among women. In contrast, sexual dysfunction was associated with increased odds of anxiety for both genders and increased odds of depression for women. Lastly, respondents of both genders who self‐identified as non‐heterosexual had higher odds of depression, and women who self‐identified as non‐heterosexual also exhibited higher odds of reporting poor or fair health. A summary of significant results is provided in Figure 2.

**TABLE 3:** Adjusted odds of poor/fair health, depressive symptoms, and anxiety symptoms according to sexuality/sexual health indicators by gender of CSF‐2023 participants: Results from multivariate logistic regression models.

		Men			Women		
		Poor/fair health	Depression	Anxiety	Poor/fair health	Depression	Anxiety
Sexual identity	Non heterosexual	1.3	**1.8**	0.9	**1.7**	**1.9**	1.1
		[0.9–1.9]	**[1.2–2.8]**	[0.5–1.6]	**[1.2–2.4]**	**[1.4–2.8]**	[0.8–2.5]
Sex in the last 12 months		0.8	0.7	1.0	**0.8**	**0.6**	**0.7**
		[0.7–1.0]	[0.5–1.1]	[0.7–1.5]	**[0.7–1.0]**	**[0.4–0.8]**	**[0.5–1.0]**
Masturbation 12 months		**0.7**	1.3	1.3	**0.7**	0.8	**0.6**
		**[0.6–0.8]**	[0.9–1.9]	[0.9–1.8]	**[0.6–0.9]**	[0.7–1.0]	**[0.5–0.8]**
Sexual dysfunction in the past 12 months^a^		1.3	1.6	**2.1**	1.2	**1.4**	**1.3**
		[0.9–1.8]	[1.0–2.6]	**[1.3–3.3]**	[0.9–1.5]	**[1.1–1.9]**	**[1.0–1.8]**
History of sexual violence	No	ref	ref	ref	ref	ref	ref
	Unwanted sexual contact	1.1	**1.7**	**1.7**	**1.3**	1.3	**1.4**
		[0.8–1.5]	**[1.2–2.9]**	**[1.1–2.6]**	**[1.0–1.5]**	[1.0–1.6]	**[1.1–1.8]**
	Sexual assault	1.3	**2.0**	**1.6**	1.1	**1.5**	1.2
		[0.9–1.7]	**[1.3–3.0]**	**[1.0–2.6]**	[1.0–1.3]	**[1.2–1.9]**	[1.0–1.5]
Sexual satisfaction	Very satisfied	ref	ref	ref	ref	ref	ref
	Somewhat satisfied	1.2	1.1	0.9	**1.2**	**1.3**	**1.3**
		[1.0–1.4]	[0.8–1.5]	[0.7–1.2]	**[1.1–1.5]**	**[1.0–1.6]**	**[1.0–1.5]**
	Unsatisfied	**2.1**	**3.0**	**2.0**	**1.8**	**2.3**	**2.0**
		**[1.7–2.7]**	**[2.1–4.3]**	**[1.4–2.9]**	**[1.5–2.2]**	**[1.7–3.0]**	**[1.5–2.6]**
Age	18–29	ref	ref	ref	ref	ref	ref
	30–39	0.9	**0.4**	0.7	**1.3**	**0.5**	**0.6**
		[0.6–1.3]	**[0.3–0.6]**	[0.5–1.2]	**[1.0–1.7]**	**[0.4–0.7]**	**[0.4–0.8]**
	40–49	1.4	**0.3**	0.8	**1.5**	**0.4**	**0.5**
		[1.0–1.8]	**[0.2–0.5]**	[0.5–1.2]	**[1.1–1.9]**	**[0.3–0.5]**	**[0.4–0.6]**
	50–59	**1.8**	**0.3**	**0.5**	**1.6**	**0.3**	**0.4**
		**[1.3–2.4]**	**[0.2–0.4]**	**[0.3–0.8]**	**[1.2–2.0]**	**[0.2–0.4]**	**[0.3–0.6]**
	60–69	**1.5**	**0.2**	**0.3**	**1.4**	**0.2**	**0.3**
		**[1.1–2.1]**	**[0.1–0.3]**	**[0.2–0.5]**	**[1.0–1.8]**	**[0.1–0.3]**	**[0.2–0.4]**
	70–89	**1.9** **[1.3–2.7]**	**0.1** **[0.1–0.2]**	**0.3** **[0.2–0.5]**	**1.8** **[1.3–2.5]**	**0.2** **[0.1–0.3]**	**0.3** **[0.2–0.4]**
Uro‐gynecological condition^b^		0.7	1.0	1.0	**1.2**	1.2	**1.2**
		[0.4–1.2]	[0.5–2.4]	[0.4–2.6]	**[1.0–1.5]**	[1.0–1.5]	**[1.0–1.5]**
Disability^c^/chronic condition^d^		**9.0**	**1.9**	**1.7**	**10.6**	**1.9**	**1.4**
		**[7.3–10.9]**	**[1.5–2.5]**	**[1.3–2.3]**	**[8.8–12.7]**	**[1.6–2.3]**	**[1.2–1.7]**
Partner with disability^e^		**1.2**	1.2	**1.6**	1.2	1.2	**1.3**
		**[1.0–1.5]**	[0.9–1.7]	**[1.2–2.3]**	[1.0–1.4]	[0.9–1.6]	**[1.0–1.7]**
Education	< High school grad	ref	ref	ref	ref	ref	ref
	High school grad	**0.7**	0.8	0.7	**0.7**	1.1	1.1
		**[0.6–0.9]**	[0.6–1.2]	[0.5–1.0]	**[0.6–0.8]**	[0.8–1.4]	[0.8–1.4]
	Bachelor	**0.8**	0.8	0.8	**0.6**	**0.8**	**0.8**
		**[0.6–0.9]**	[0.6–1.1]	[0.6–1.2]	**[0.5–0.7]**	**[0.6–1.0]**	**[0.6–1.0]**
	Graduate education	**0.5**	**0.7**	**0.6**	**0.4**	**0.7**	0.8
		**[0.4–0.6]**	**[0.5–1.0]**	**[0.4–0.9]**	**[0.3–0.5]**	**[0.5–0.9]**	[0.7–1.1]
Health insurance	National + private	ref	ref	ref	ref	ref	ref
	National plan only	1.1	1.3	**2.8**	1.1	1.3	0.9
		[0.8–1.6]	[0.7–2.4]	**[1.5–5.0]**	[0.7–1.7]	[0.7–2.2]	[0.5–1.6]
	Low‐income public coverage or no coverage income/no plan	**2.2**	1.5	1.3	**1.5**	1.0	1.1
		**[1.5–3.2]**	[0.8–2.6]	[0.8–2.3]	**[1.1–2.1]**	[0.6–1.6]	[0.7–1.8]
	Other	1.4	1.6	1.6	1.0	1.3	2.2
		[0.6–3.3]	[0.6–4.5]	[0.5–4.9]	[0.3–3.2]	[0.4–4.3]	[0.5–9.6]
Facing financial difficulty		**1.9** **[1.6–2.3]**	**1.8** **[1.4–2.3]**	**1.5** **[1.1–2.0]**	**1.8** **[1.6–2.2]**	**1.6** **[1.3–2.0]**	**1.8** **[1.5–2.1]**
Foreign born		1.0	0.7	0.5	1.0	1.1	0.8
		[0.7–1.5]	[0.4–1.5]	[0.2–1.1]	[0.7–1.4]	[0.6–1.7]	[0.5–1.3]
Residence	Large city/suburb	ref	ref	ref	ref	ref	ref
	Medium/small size city	1.1	1.1	0.8	1.0	1.1	0.9
		[0.9–1.4]	[0.8–1.5]	[0.6–1.2]	[0.8–1.1]	[0.9–1.4]	[0.7–1.2]
	Rural	1.0	1.1	1.3	1.1	1.0	0.9
		[0.8–1.2]	[0.8–1.5]	[0.9–1.8]	[0.9–1.3]	[0.7–1.2]	[0.7–1.1]

*Note:* All bold associations are statistically significant with *p* values < 0.05. CI are more informative than *p* values and indicate the significance.

^a^ Sexual dysfunction (last 12 months): at least one sexual problem lasting 6 months or more and causing distress.

^b^ Uro‐gynecological condition: prostate‐related problems in men and breast‐, ovary‐, or uterus‐related problems in women.

^c^ Functional disability: limitation in daily activities for at least 6 months due to a health problem.

^d^ Chronic illness: chronic illness or health problem lasting at least 6 months.

^e^ Partner disability: partner reported to have limitation in daily activities for at least 6 months due to a health problem.

**FIGURE 2:**
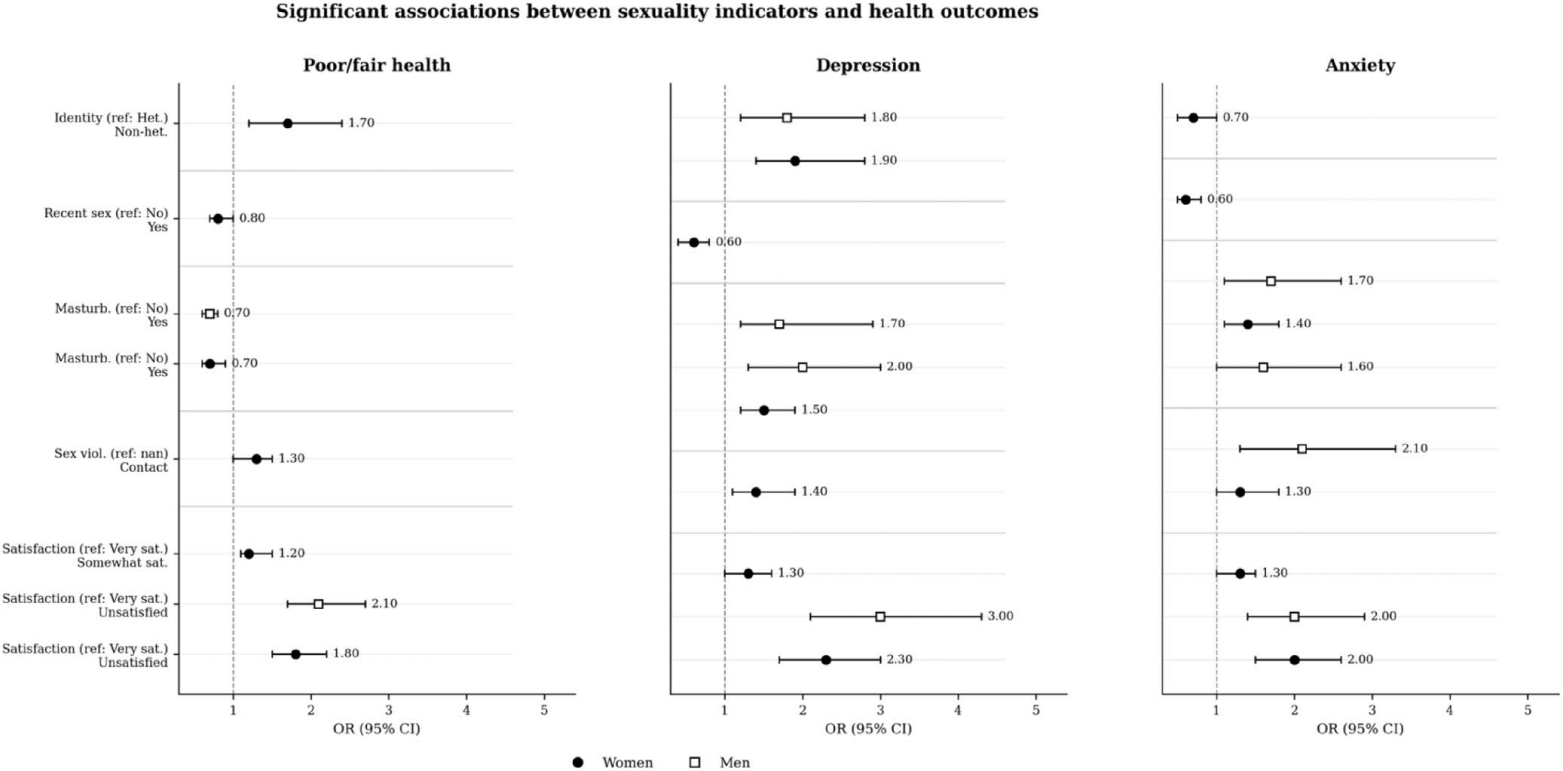
Summary of statistically significant multivariate results related to associations between indicators of sexuality and health outcomes among CSF‐2023 participants.

Other factors consistently linked to increased odds of all health conditions included having a disability or chronic illness and experiencing financial difficulty. In contrast, higher education was associated with decreased odds of all conditions, except for anxiety in women. Age increased the odds of reporting poor or fair health but decreased the odds of mental health issues for both genders. Gynecological conditions raised the odds of poor or fair health and anxiety among women. Moreover, caring for a disabled partner increased the odds of anxiety for both genders and the odds of poor/fair health among men (Table 3).

### 3.3 Differential Associations by Age, Partnership Status, Health Status, and Economic Instability

A stratified analysis by age groups (18–30 years, 30–59 years, and 60–89 years) revealed significant variations in the relationship between sexuality and health conditions throughout the life course (Appendix 1). Notably, the link between sexual violence and poor/fair health was more pronounced in respondents under 30 compared to older individuals. Sexual activity was more strongly associated with decreased odds of depression in men and anxiety in women for respondents over 60, relative to younger participants. At the same time, masturbation was associated with greater reductions in the odds of anxiety in women over 60 compared to women less than 30. Additionally, sexual dysfunction increased the odds of depression only in men aged 30–59. Lastly, the associations between sexual satisfaction and poor or fair health and anxiety were stronger in women under 60 than in those 60 and older.

Stratified analysis by partnership status, and by sexual identity revealed few differential associations (Appendix 2 & 3). Sexual activity only decreased the odds of depression and anxiety for partnered women but not for non‐partnered women. Likewise, sexual dissatisfaction only increased the odds of anxiety for partnered women but not for non‐partnered women (Appendix 2). Additionally, sexual dissatisfaction was associated with greater increases in the odds of poor/health among non‐heterosexual men and higher increases in the odds of depression for non‐heterosexual women, while unwanted sexual contact only increased the odds of anxiety among heterosexual women (Appendix 3).

There were few differences in associations according to the respondent's health status (functional disability or chronic illness), apart from masturbation, which only reduced the odds of depression among unhealthy women (Appendix 4). Likewise, economic instability had little bearing on the associations of interest except for unwanted sexual contact, which only increased the odds of poor/fair health among women reporting economic instability (Appendix 5).

## 4 Discussion

This study highlights the intricate relationship between sexuality and health, demonstrating nuanced variations by age and gender. Across diverse demographic groups, consistent correlations were found between sexual dissatisfaction and poor physical and mental health. Associations between other sexuality indicators (such as recent sexual activity, sexual dysfunction, and sexual violence) and health outcomes varied by type of health outcome (self‐perceived health, depression, or anxiety) and subgroup of the population (gender, age, or partnered individuals). These findings remained robust after adjustments for a broad array of sociodemographic factors and disability conditions.

Our findings of a positive association between sexual activity, sexual satisfaction, and improved physical or mental health are consistent with previous studies in other settings [42, 43]. These associations are bi‐directional and cyclical, as Graugaard notes [2]. Sexual issues can trigger negative emotions, further deteriorating mental health and undermining both motivation and the ability to manage chronic illnesses [2]. Regardless of directionality, these results underscore the need to include sexual health in routine clinical assessment, with attention to various aspects of sexuality, including sexual satisfaction. In our study, sexual dissatisfaction, reported by 17% of women and 21% of men, was more strongly and consistently linked to poor physical and mental health than any other sexuality indicator, even when controlling for sexual dysfunction, sexual activity, and adverse sexual experiences such as sexual violence. As such, sexual satisfaction should be considered as an integral component of overall health and well‐being.

From an epidemiological perspective, age confounds the association between sexuality and health due to its association with declining partnerships, reduced sexual functioning [44], and increased prevalence of health conditions. However, few studies have explored how age moderates the interplay between sexuality and health. Our findings provide evidence of changes in these associations over the life course: sexual violence had more pronounced negative health effects on younger respondents, while sexual activity and satisfaction had stronger positive correlations with mental health in older respondents. Two studies in the US have also reported differential associations by age. In one study, the effect of erectile dysfunction on depressive symptoms was greater among men aged 50–59 than those aged 60–69 years [28]. The second study found stronger links between sexual satisfaction and health conditions among younger participants, specifically noting correlations with hypertension in young women and depression or anxiety in young men [18]. Age‐related differences in our study were not explained by disability or chronic conditions, which increased with age but had little impact on the examined associations. Similarly, changes in the correlation between sexuality and health changed little according to partnership status, which declined with age but minimally affected the observed associations. One notable exception was that sexual activity in the past year had a more positive association with mental health among partnered women compared to their unpartnered counterparts.

Gender had a modest effect on the associations between sexuality and health in our study. Masturbation was linked to a reduction in poor/fair health and anxiety among women but an increase in mental health symptoms for men in certain age groups (albeit non‐significant). Other studies in the US [18, 45, 46], or Germany [16]; showed no gender differences in associations between sexuality and health, while a British study found no interaction for the association between overall self‐rated health and sexuality but identified gender differences in associations according to specific health conditions [47].

We also found limited evidence of intersectional effects involving gender, sexual orientation, or economic instability, despite higher physical and mental morbidity observed in these populations. To date, one study has explored differential associations between sexuality and health across gender and race/ethnicity in the US, finding no significant interactions [18]. Despite these results, we still believe integrating intersectionality with a life course perspective is valuable [48], given robust evidence of compounded sexual health disadvantages among multi‐minoritized populations [49] (according to race/ethnicity, social class, sexual and gender identity, etc.), and the intersecting effects of social hierarchies on aging [50]. Such an approach is essential in reducing health inequities.

Our results should be interpreted with several limitations in mind. First, the cross‐sectional design of the study prevents any causal inferences, as most associations are bi‐directional, requiring a focus on correlations rather than causation. Second, our assessment of sexual satisfaction could be enhanced to better capture how physical, emotional, and relational aspects of sexual experiences [21] relate to broader constructs of health and well‐being. Third, our assessment of gender was limited to a single item asked at study entry, in which respondents identified as men, women, or another category. This approach did not adequately capture gender diversity, as only five participants identified outside the categories of men or women. Fourth, the sample size limits the statistical power of interaction tests and the interpretation of intersectionality effects, highlighting the need for further research using advanced statistical techniques to better capture between‐group and within‐group heterogeneity [51]. Additionally, a low response rate may introduce selection bias and limit the generalizability of our findings, although careful application of post‐stratification weights helps correct for non‐response bias. Finally, the study did not include couples, limiting our understanding of the relational context shaping sexual practices and experiences.

Despite these limitations, we believe our results contribute to the literature exploring positive links between sexuality and health across the life course. Our large nationally representative survey encompasses a wide age range, allowing for an intersectional approach to the understanding of sexuality and health over the life course. The detailed information on sexuality enables a thorough examination of correlations between sexual practices and health, controlling for a comprehensive array of confounders, which enhances the robustness of our findings.

These findings have implications for public health policy, clinical practice, and future research. They support moving beyond a narrow focus on sexual risk prevention to recognize sexual well‐being as part of overall health across the life course. In practice, this suggests integrating a brief assessment of sexual well‐being, including sexual satisfaction, into primary care and general health services, not only specialist SRH settings, and supporting this through training and task sharing with frontline providers. Our results also point to the need for more life‐course‐sensitive approaches, with attention to the health consequences of sexual violence in younger populations and to sexuality as part of healthy aging in older adults. More broadly, they suggest that sexuality may serve not only as a site of prevention, but also as a marker of general physical and mental health. Future research should extend this work through longitudinal designs that can better disentangle the bidirectional relationship between sexuality and health and clarify the mechanisms linking them, to inform more effective public health and clinical interventions.

## 5 Conclusion

In this nationally representative study, we examined the associations between multiple dimensions of sexuality and self‐reported general and mental health across the life course, with particular attention to gender disparities. Although the strength and direction of these associations varied across population groups and across dimensions of sexuality and health, sexual dissatisfaction emerged as the most consistent correlate of poor physical and mental health. These findings suggest that the quality of sexual life should be understood as an integral component of overall health and well‐being, rather than as a peripheral concern. For policymakers and practitioners, the results support moving beyond a narrow focus on sexual risk prevention to incorporate sexual well‐being into public health and primary care approaches across the life course. For researchers, the findings underscore the importance of examining sexuality and health together through life course and intersectional perspectives, and of further investigating the bidirectional pathways and mechanisms linking sexual experiences to broader health outcomes.

## Appendix 1 Odds of Poor/Fair Health, Depression or Anxiety by Sexuality Indicators According to Age and Gender: Results From Multivariate Stratified Analysis

**Table:**

Sexuality indicator	Poor/fair general health						Depression					
	Men			Women			Men			Women		
	18–29	30–59	60–89	18–29	30–59	60–89	18–29	30–59	60–89	18–29	30–59	60–89
Non heterosexual identity	1.5 [0.8–2.8]	**1.8 [1.2–2.9]**	0.6 [0.3–1.3]	**1.9 [1.1–3.4]**	**1.5 [1.0–2.3]**	1.5 [0.5–4.0]	1.5 [0.7–3.2]	1.2 [0.7–2.2]	**4.1 [1.6–10.6]**	**1.7 [1.0–2.7]**	**2.2 [1.2–3.7]**	1.4 [0.4–4.6]
Sex in last 12 months	0.8 [0.4–1.8]	1.0 [0.7–1.3]	0.8 [0.6–1.1]	0.7 [0.3–1.7]	0.8 [0.6–1.1]	0.8 [0.6–1.1]	1.0 [0.5–2.1]	1.0 [0.6–1.5]	**0.3 [0.2–0.7]**	1.0 [0.4–2.0]	**0.5 [0.4–0.9]**	**0.5 [0.3–0.8]**
Masturbation 12 months	0.7 [0.3–1.6]	**0.6 [0.5–0.8]**	**0.7 [0.5–1.0]**	**0.5 [0.4–0.8]**	**0.8 [0.7–1.0]**	**0.7 [0.5–0.9]**	0.7 [0.2–2.9]	1.2 [0.7–2.0]	**1.9 [1.1–3.3]**	0.8 [0.5–1.4]	**0.8 [0.6–1.0]**	0.8 [0.5–1.3]
Sexual dysfunction	1.7 [0.5–5.9]	1.5 [0.9–2.5]	1.0 [0.6–1.7]	0.8 [0.5–1.4]	**1.4 [1.0–1.9]**	1.0 [0.6–1.7]	0.6 [0.2–1.9]	**2.5 [1.3–4.6]**	1.2 [0.4–3.2]	1.2 [0.7–2.1]	**1.4 [1.0–1.9]**	1.9 [0.8–4.5]
Unwanted sexual contact	2.1 [0.8–5.3]	0.9 [0.6–1.4]	1 [0.6–1.8]	**2.3 [1.4–3.9]**	1.2 [0.9–1.6]	1.1 [0.8–1.6]	1.8 [0.7–4.6]	**2.1 [1.2–3.8]**	1.2 [0.4–3.2]	1.3 [0.8–2.3]	**1.4 [1.0–2.0]**	1.1 [0.6–2.0]
Sexual assault	**2.3 [1.3–4.3]**	0.7 [0.5–1.1]	1.9 [0.9–3.8]	**1.6 [1.0–2.5]**	1.1 [0.9–1.3]	1.2 [0.8–1.6]	**2.8 [1.2–6.2]**	1.5 [0.8–2.8]	2.8 [0.9–8.3]	**2 [1.3–3.0]**	**1.4 [1.1–1.8]**	1.6 [0.9–2.6]
Somewhat dissatisfied with sexual life	0.7 [0.5–1.4]	**1.4 [1.1–1.8]**	1.1 [0.8–1.6]	1.1 [0.7–1.7]	1.0 [0.8–1.3]	**1.6 [1.2–2.1]**	1.0 [0.6–1.9]	1.0 [0.6–1.5]	1.2 [0.6–2.3]	**1.9 [1.2–2.8]**	1.2 [0.8–1.6]	1.1 [0.7–1.8]
Unsatisfied with sexual life	1.2 [0.6–2.4]	**2.1 [1.6–2.8]**	**2.7 [1.8–3.8]**	**2.8 [1.5–5.1]**	**1.6 [1.2–2.0]**	**2.0 [1.4–2.8]**	**3.0 [1.5–6.0]**	**3.6 [2.3–5.8]**	**2.2 [1.0–4.9]**	**4.0 [2.2–7.0]**	**2.8 [2.0–3.9]**	1.3 [0.7–2.4]

Sexuality indicator	Anxiety					
	Men			Women		
	18–29	30–59	60–89	18–29	30–59	60–89
Non heterosexual identity	0.7 [0.2–1.9]	0.9 [0.5–1.8]	**2.9 [1.0–8.6]**	**2.5 [1.5–4.1]**	1.0 [0.5–2.0]	1.3 [0.4–4.3]
Sex in last 12 months	0.5 [0.2–1.3]	1.4 [0.8–2.5]	1.2 [0.5–2.4]	2.1 [0.8–5.7]	**0.7 [0.5–0.9]**	**0.6 [0.4–1.0]**
Masturbation 12 months	0.9 [0.2–3.4]	**1.6 [1.0–2.5]**	1 [0.6–1.8]	0.9 [0.6–1.4]	**0.6 [0.5–0.8]**	**0.4 [0.2–0.6]**
Sexual dysfunction	1.4 [0.3–6.4]	**2.7 [1.5–4.7]**	1.5 [0.6–3.8]	0.8 [0.5–1.5]	**1.5 [1.1–2.1]**	1.0 [0.4–2.2]
Unwanted sexual contact	1.7 [0.7–4.5]	1.5 [0.9–1.8]	**2.6 [1.1–6.1]**	1.5 [0.9–2.6]	**1.6 [1.2–2.2]**	1.2 [0.7–2.1]
Sexual assault	1.4 [0.5–3.7]	1.6 [0.9–3.0]	2.6 [0.8–8.9]	1.3 [0.9–2.0]	1.2 [0.9–1.6]	1.3 [0.8–2.2]
Somewhat dissatisfied with sexual life	1.2 [0.6–2.4]	0.8 [0.5–1.2]	1.1 [0.5–2.4]	1.4 [0.9–2.1]	**1.3 [1.0–1.7]**	1.0 [0.7–1.6]
Unsatisfied with sexual life	**2.3 [1.0–5.2]**	**1.8 [1.2–2.9]**	**3.0 [1.4–6.9]**	**3.3 [1.9–5.9]**	**2.5 [1.8–3.5]**	1.1 [0.7–2.0]

*Note:* Bold numbers indicate statistical significance at *p* < 0.05. Red numbers indicate a statistically significant interaction by the stratifying variable at *p* < 0.05.

## Appendix 2 Odds of Poor/Fair Health, Depression or Anxiety by Sexuality Indicators According to Partnership Status and Gender: Results From Multivariate Stratified Analysis

**Table:**

Sexuality indicator	Poor/fair general health				Depression				Anxiety			
	Men		Women		Men		Women		Men		Women	
	Partner	No partner	Partner	No partner	Partner	No partner	Partner	No partner	Partner	No partner	Partner	No partner
Non heterosexual identity	1.1 [0.7–1.8]	1.3 [0.7–2.3]	**1.6 [1.1–2.3]**	**2.4 [1.2–4.8]**	1.3 [0.7–2.2]	**2.1 [1.0–4.3]**	**1.9 [1.2–3.0]**	**2.0 [1.1–3.6]**	0.9 [0.4–1.6]	1.0 [0.4–2.4]	**1.5 [1.0–2.3]**	**2.7 [1.4–5.0]**
Sex in last 12 months	0.8 [0.6–1.1]	1.0 [0.6–1.4]	0.8 [0.6–1.1]	1.0 [0.7–1.4]	0.8 [0.4–1.5]	1.1 [0.7–1.8]	**0.4 [0.3–0.7]**	1.0 [0.6–1.5]	1.1 [0.6–2.1]	1.1 [0.6–2.0]	**0.4 [0.3–0.6]**	1.4 [0.9–2.2]
Masturbation 12 months	**0.7 [0.5–0.8]**	0.7 [0.5–1.1]	**0.8 [0.7–0.9]**	**0.6 [0.4–0.8]**	1.2 [0.8–1.9]	1.3 [0.7–2.6]	0.8 [0.7–1.1]	0.8 [0.5–1.2]	**1.4 [1.0–2.1]**	1.1 [0.6–2.2]	**0.7 [0.5–0.8]**	**0.6 [0.4–0.9]**
Sexual dysfunction	1.2 [0.8–1.8]	1.3 [0.6–2.8]	1.1 [0.8–1.4]	**2.2 [1.0–4.6]**	**2.3 [1.3–3.9]**	0.6 [0.2–1.9]	**1.5 [1.0–2.1]**	0.9 [0.5–1.8]	**2.0 [1.2–3.3]**	2.6 [0.9–8.1]	**1.4 [1.0–1.8]**	0.9 [0.5–1.9]
Unwanted sexual contact	1.1 [0.7–1.6]	0.9 [0.5–1.8]	1.2 [0.9–1.5]	**1.6 [1.1–2.3]**	**1.8 [1.1–2.9]**	1.4 [0.6–3.3]	1.2 [0.9–1.7]	1.2 [0.7–2.1]	**2.0 [1.2–3.3]**	1 [0.3–2.8]	**1.3 [1.0–1.8]**	1.4 [0.8–2.5]
Sexual assault	1.2 [0.8–1.7]	1.4 [0.8–2.4]	1.1 [0.9–1.3]	1.2 [0.9–1.7]	**1.8 [1.0–3.2]**	**2.6 [1.3–5.2]**	**1.4 [1.1–1.8]**	**1.7 [1.1–2.5]**	1.7 [0.9–3.0]	1.5 [0.7–3.6]	1.0 [0.8–1.3]	**1.6 [1.1–2.5]**
Somewhat dissatisfied with sexual life	1.2 [0.9–1.4]	1.2 [0.7–2.0]	**1.4 [1.2–1.7]**	1.0 [0.7–1.4]	0.9 [0.7–1.3]	1.4 [0.6–3.0]	**1.3 [1.0–1.7]**	1.1 [0.7–1.9]	0.9 [0.7–1.3]	0.9 [0.4–2.0]	**1.5 [1.1–1.9]**	0.8 [0.5–1.3]
Unsatisfied with sexual life	**2.5 [1.9–3.3]**	1.5 [0.9–2.4]	**1.9 [1.5–2.5]**	**1.6 [1.1–2.4]**	**2.4 [1.6–3.8]**	**3.7 [1.8–7.7]**	**2.6 [1.8–3.7]**	1.6 [0.9–2.7]	**2.3 [1.5–3.5]**	1.8 [0.8–3.9]	**2.3 [1.7–3.2]**	1.3 [0.8–2.1]

*Note:* Bold numbers indicate statistical significance at *p* < 0.05. Red numbers indicate a statistically significant interaction by the stratifying variable at *p* < 0.05.

## Appendix 3 Odds of Poor/Fair Health, Depression or Anxiety by Sexuality Indicators According to Sexual Self‐Identity and Gender: Results From Multivariate Stratified Analysis

**Table:**

Sexuality indicator	Poor/fair general health				Depression				Anxiety			
	Men		Women		Men		Women		Men		Women	
	Heterosexual	Non‐heterosexual	Heterosexual	Non‐heterosexual	Heterosexual	Non‐heterosexual	Heterosexual	Non‐heterosexual	Heterosexual	Non‐heterosexual	Heterosexual	Non‐heterosexual
Sex in last 12 months	**0.8 [0.6–1.0]**	1.4 [0.7–2.9]	**0.8 [0.7–1.0]**	1.4 [0.7–2.8]	**0.7 [0.5–1.0]**	0.6 [0.2–1.7]	**0.6 [0.5–0.8]**	0.6 [0.2–1.4]	1.1 [0.7–1.6]	1.2 [0.4–3.3]	**0.8 [0.6–1.0]**	0.7 [0.3–1.8]
Masturbation 12 months	**0.7 [0.5–0.8]**	0.6 [0.3–1.1]	**0.7 [0.6–0.9]**	0.7 [0.4–1.3]	1.1 [0.8–1.6]	**3.6 [1.2–11.2]**	**0.8 [0.7–1.0]**	1.0 [0.4–2.3]	**1.4 [1.0–1.9]**	1.8 [0.5–5.9]	**0.6 [0.5–0.8]**	0.6 [0.3–1.4]
Sexual dysfunction	1.3 [0.9–1.8]	1.0 [0.3–2.9]	1.2 [0.9–1.6]	0.8 [0.3–1.7]	**1.7 [1.1–2.7]**	0.6 [0.1–4.0]	**1.4 [1.1–1.9]**	1.1 [0.4–3.0]	**2.2 [1.4–3.6]**	0.5 [0.1–4.2]	**1.3 [1.0–1.7]**	1.2 [0.5–3.1]
Unwanted sexual contact	1.1 [0.7–1.5]	0.9 [0.4–2.1]	**1.3 [1.0–1.5]**	1.2 [0.7–2.3]	**1.6 [1.0–2.4]**	**3.0 [1.1–8.3]**	**1.4 [1.1–1.8]**	0.8 [0.4–1.7]	**1.7 [1.1–2.7]**	0.6 [0.1–5.6]	**1.5 [1.2–2.0]**	0.7 [0.3–1.6]
Sexual assault	1.2 [0.9–1.7]	1.1 [0.6–2.2]	**1.2 [1.0–1.4]**	0.9 [0.5–2.1]	**1.9 [1.2–2.8]**	1.6 [0.7–3.7]	**1.6 [1.3–1.9]**	1.4 [0.8–2.7]	**1.8 [1.0–3.0]**	1.3 [0.4–4.2]	**1.2 [1.0–1.5]**	1.6 [0.8–3.3]
Somewhat dissatisfied with sexual life	**1.2 [1.0–1.4]**	1.4 [0.8–2.5]	**1.2 [1.0–1.5]**	1.4 [0.8–2.3]	1.1 [0.8–1.4]	**3.2 [1.5–7.3]**	**1.3 [1.0–1.6]**	**2.2 [1.2–4.3]**	0.9 [0.6–1.2]	**3.5 [1.0–11.7]**	**1.2 [1.0–1.6]**	1.4 [0.7–2.7]
Unsatisfied with sexual life	**2.2 [1.8–2.8]**	1.7 [0.8–3.7]	**1.7 [1.3–2.0]**	**3.9 [1.9–8.0]**	**3.0 [2.1–4.2]**	**2.8 [1.0–7.8]**	**2.4 [1.8–3.1]**	**3.2 [1.5–7.2]**	**1.9 [1.3–2.8]**	**6.8 [1.9–25.0]**	**2.1 [1.6–2.7]**	**2.3 [1.0–5.3]**

*Note:* Bold numbers indicate statistical significance at *p* < 0.05. Red numbers indicate a statistically significant interaction by the stratifying variable at *p* < 0.05.

## Appendix 4 Odds of Poor/Fair Health, Depression or Anxiety by Sexuality Indicators According to Health Status and Gender: Results From Multivariate Stratified Analysis

**Table:**

Sexuality indicator	Poor/fair general health				Depression				Anxiety			
	Men		Women		Men		Women		Men		Women	
	Healthy	Unhealthy	Healthy	Unhealthy	Healthy	Unhealthy	Healthy	Unhealthy	Healthy	Unhealthy	Healthy	Unhealthy
Non heterosexual identity	0.9 [0.4–1.2]	**1.5 [1.0–2.3]**	1.8 [0.9–3.4]	**1.7 [1.2–2.4]**	1.7 [0.9–3.3]	**2.0 [1.1–3.6]**	1.2 [0.7–2.1]	**2.7 [1.7–4.2]**	0.7 [0.3–1.6]	1.2 [0.6–2.6]	1.2 [0.8–2.0]	**2.3 [1.4–3.8]**
Sex in last 12 months	1.2 [0.7–2.1]	**0.7 [0.6–1.0]**	0.9 [0.6–1.5]	**0.8 [0.6–1.0]**	1.1 [0.6–1.9]	**0.6 [0.4–0.9]**	0.7 [0.4–1.1]	**0.5 [0.4–0.7]**	1.5 [0.8–2.8]	0.9 [0.5–1.5]	0.8 [0.5–1.2]	**0.7 [0.5–0.9]**
Masturbation 12 months	0.8 [0.5–1.2]	**0.7 [0.5–0.8]**	0.9 [0.6–1.3]	**0.7 [0.6–0.8]**	1.0 [0.5–1.9]	**1.5 [1.0–2.3]**	1.1 [0.8–1.5]	**0.7 [0.6–0.9]**	1.7 [0.9–3.2]	1.2 [0.8–1.8]	0.8 [0.6–1.1]	**0.6 [0.5–0.7]**
Sexual dysfunction	1.3 [0.6–3.0]	1.3 [0.9–1.8]	0.9 [0.4–1.8]	1.2 [0.9–1.6]	1.6 [0.7–3.7]	1.7 [0.9–3.1]	1.1 [0.7–1.7]	**1.6 [1.1–2.4]**	1.7 [0.7–3.7]	**2.4 [1.4–4.2]**	1.1 [0.7–1.6]	**1.6 [1.1–2.3]**
Unwanted sexual contact	1.4 [0.7–3.0]	1 [0.7–1.4]	1.1 [0.7–1.9]	**1.3 [1.0–1.6]**	**2.6 [1.4–5.1]**	1.2 [0.7–2.1]	**1.7 [1.1–2.5]**	1.1 [0.8–1.5]	1.1 [0.5–2.4]	**2.1 [1.3–3.5]**	**1.4 [1.0–2.0]**	**1.4 [1.0–2.0]**
Sexual assault	1.2 [0.6–2.4]	1.3 [0.9–1.8]	1.0 [0.7–1.5]	**1.2 [1.0–1.4]**	**2.3 [1.3–4.1]**	**1.9 [1.0–3.6]**	**1.7 [1.2–2.3]**	**1.4 [1.1–1.9]**	**2.2 [1.1–4.2]**	1.2 [0.6–2.5]	1.1 [0.8–1.6]	1.2 [0.9–1.6]
Somewhat dissatisfied with sexual life	1.5 [0.7–2.3]	1.1 [0.9–1.4]	1.1 [0.8–1.7]	**1.3 [1.1–1.5]**	0.8 [0.5–1.3]	1.4 [0.9–2.1]	**1.6 [1.1–2.2]**	1.1 [0.8–1.6]	0.8 [0.5–1.3]	1 [0.7–1.6]	**1.3 [1.0–1.8]**	1.2 [0.9–1.6]
Unsatisfied with sexual life	**2.4 [1.5–3.9]**	**2.0 [1.6–0.02.6]**	**1.6 [1.0–2.5]**	**1.8 [1.5–2.3]**	**3.3 [1.9–5.5]**	**3.0 [1.9–4.9]**	**3.2 [2.1–4.9]**	**1.9 [1.4–2.8]**	**2.1 [1.2–3.5]**	**2.0 [1.2–3.3]**	**2.2 [1.5–3.3]**	**1.9 [1.3–2.7]**

*Note:* Bold numbers indicate statistical significance at *p* < 0.05. Red numbers indicate a statistically significant interaction by the stratifying variable at *p* < 0.05.

## Appendix 5 Odds of Poor/Fair Health, Depression or Anxiety by Sexuality Indicators According to Financial Situation and Gender: Results From Multivariate Stratified Analysis

**Table:**

Sexuality indicator	Poor/fair general health				Depression				Anxiety			
	Men		Women		Men		Women		Men		Women	
	Financial problem	No financial problem	Financial problem	No financial problem	Financial problem	No financial problem	Financial problem	No financial problem	Financial problem	No financial problem	Financial problem	No financial problem
Non heterosexual identity	**1.6 [1.0–2.6]**	1.1 [0.6–1.9]	**2.0 [1.2–3.4]**	**1.4 [1.1–3.1]**	**2.3 [1.1–4.4]**	1.4 [0.8–2.5]	**1.8 [1.1–3.0]**	**1.9 [1.2–3.1]**	0.6 [0.3–1.6]	1.3 [0.7–2.4]	**1.7 [1.0–2.9]**	**1.7 [1.1–2.6]**
Sex in last 12 months	1.0 [0.7–1.3]	**0.7 [0.5–1.0]**	0.8 [0.6–1.1]	0.9 [0.7–1.1]	1.0 [0.6–1.8]	**0.5 [0.3–0.8]**	**0.6 [0.4–0.9]**	**0.5 [0.4–0.8]**	1 [0.6–1.7]	1.1 [0.6–1.9]	0.8 [0.5–1.2]	**0.6 [0.4–0.8]**
Masturbation 12 months	**0.6 [0.5–0.8]**	**0.7 [0.6–0.9]**	**0.7 [0.6–0.9]**	**0.7 [0.6–0.9]**	1.3 [0.8–2.2]	1.2 [0.7–2.1]	0.8 [0.6–1.1]	0.9 [0.7–1.1]	1.2 [0.7–1.9]	**1.7 [1.0–2.6]**	**0.6 [0.5–0.8]**	**0.7 [0.6–0.9]**
Sexual dysfunction	1.3 [0.8–2.1]	1.2 [0.8–1.9]	1. [0.7–1.5]	1.2 [0.9–1.7]	1.6 [0.8–3.3]	**1.8 [1.0–3.3]**	1.2 [0.8–1.8]	**1.6 [1.1–2.4]**	**2.9 [1.5–5.8]**	1.5 [0.8–2.7]	1.1 [0.7–1.6]	**1.7 [1.2–2.4]**
Unwanted sexual contact	0.8 [0.5–1.4]	1.4 [0.9–2.0]	**1.7 [1.2–2.3]**	1.0 [0.8–1.3]	**2.4 [1.3–4.4]**	1.3 [0.7–2.4]	1.3 [0.9–2.0]	1.3 [0.9–1.8]	**2.1 [1.1–3.9]**	1.6 [0.9–2.9]	**1.6 [1.2–2.4]**	1.2 [0.9–1.6]
Sexual assault	1.2 [0.8–1.8]	1.3 [0.8–2.0]	1.2 [0.9–1.5]	1.1 [0.8–1.3]	**2.0 [1.1–3.7]**	**2.1 [1.2–3.8]**	**1.5 [1.1–2.0]**	**1.6 [1.2–2.1]**	1.7 [0.8–3.3]	**1.9 [1.0–3.5]**	1.2 [0.9–1.6]	1.2 [0.9–1.6]
Somewhat dissatisfied with sexual life	1.2 [0.9–1.6]	1.2 [0.9–1.5]	**1.3 [1.0–1.6]**	**1.3 [1.0–1.6]**	1.0 [0.6–1.6]	1.1 [0.7–1.7]	1.2 [0.9–1.7]	**1.4 [1.0–1.9]**	1 [0.6–1.5]	0.9 [0.6–1.4]	1.1 [0.8–1.5]	**1.5 [1.1–1.9]**
Unsatisfied with sexual life	**2.3 [1.6–3.2]**	**2 [1.5–2.7]**	**1.8 [1.3–2.4]**	**1.9 [1.5–2.5]**	**3.1 [1.9–5.2]**	**2.8 [1.7–4.7]**	**2.7 [1.8–4.0]**	**2 [1.4–2.9]**	**2.1 [1.3–3.6]**	**1.9 [1.1–3.3]**	**2.1 [1.5–3.1]**	**1.8 [1.3–2.6]**

*Note:* Bold numbers indicate statistical significance at *p* < 0.05. Red numbers indicate a statistically significant interaction by the stratifying variable at *p* < 0.05.
